# Biosensor Technologies in Medicine: from Detection of Biochemical Markers to Research into Molecular Targets (Review)

**DOI:** 10.17691/stm2020.12.6.09

**Published:** 2020-12-28

**Authors:** B.G. Andryukov, I.N. Lyapun, E.V. Matosova, L.M. Somova

**Affiliations:** Leading Researcher, Laboratory of Molecular Microbiology; G.P. Somov Institute of Epidemiology and Microbiology, 1 Selskaya St., Vladivostok, 690087, Russia; Researcher, Laboratory of Molecular Microbiology; G.P. Somov Institute of Epidemiology and Microbiology, 1 Selskaya St., Vladivostok, 690087, Russia; Junior Researcher, Laboratory of Molecular Microbiology; G.P. Somov Institute of Epidemiology and Microbiology, 1 Selskaya St., Vladivostok, 690087, Russia; Professor, Chief Researcher, Laboratory of Molecular Microbiology G.P. Somov Institute of Epidemiology and Microbiology, 1 Selskaya St., Vladivostok, 690087, Russia

**Keywords:** biosensors, label-free biosensor, laboratory diagnostics, infectious diseases, sensory strategies, molecular markers

## Abstract

Infections are a major cause of premature death. Fast and accurate laboratory diagnostics of infectious diseases is a key condition for the timely initiation and success of treatment. Potentially, it can reduce morbidity, as well as prevent the outbreak and spread of dangerous epidemics. The traditional methods of laboratory diagnostics of infectious diseases are quite time- and labour-consuming, require expensive equipment and trained personnel, which is crucial within limited resources. The fast biosensor-based methods that combine the diagnostic capabilities of biomedicine with modern technological advances in microelectronics, optoelectronics, and nanotechnology make an alternative.

The modern achievements in the development of label-free biosensors make them promising diagnostic tools that combine rapid detection of specific molecular markers, simplicity, ease-of-use, efficiency, accuracy, and cost-effectiveness with the tendency to the development of portable platforms. These qualities exceed the generally accepted standards of microbiological and immunological diagnostics and open up broad prospects for using these analytical systems in clinical practice directly at the site of medical care provision (point-of-care, POC concept).

A wide variety of modern biosensor designs are based on the use of diverse formats of analytical and technological strategies, identification of various regulatory and functional molecular markers associated with infectious pathogens. The solution to the existing problems in biosensing will open up great prospects for these rapidly developing diagnostic biotechnologies.

## Introduction

The most terrible tragedies of mankind in recent centuries have been associated with the outbreaks and spread of pandemic infections that have claimed hundreds of millions of lives. Despite the obvious success of the global health care system, the risk of epidemics of known, new, and recurring infections remains a serious threat to the world’s population. The bacterial and viral infectious diseases with the fecal-oral mechanism of infection claim about 2 million lives annually. The recent outbreaks of Ebola, Zika, Dengue, Middle East respiratory syndrome, severe acute respiratory syndrome, and H5N1 influenza, as well as the increasing resistance of bacteria to antimicrobial drugs, increase the urgency of searching for new effective diagnostic tools aimed at early and rapid detection of pathogens [1–3].

With broadening our knowledge on the complex biochemical processes underlying the pathogenesis of infectious processes, it has become necessary to develop more sensitive and highly specific diagnostic strategies. They are based on the identification of molecular markers, profiling of microorganisms without cultivation, enrichment, and isolation of pure cultures. These methods will become ideal analytical tools for controlling pathogenic microorganisms and make a basis for identifying the relationship between molecular structures and biological processes [1, 4, 5].

Classical microbiological and immunoserological methods, as well as modern diagnostic platforms such as ELISA and chemiluminescence analysis, PCR, flow cytometry, and mass spectrometry (MALDI) adopted in recent decades, prevail when accurate verification of infectious agents is needed in centralized laboratories of medical hospitals and centers. However, these diagnostic tools require expensive equipment, long testing times, and qualified personnel, and are not always available for small hospitals, especially under limited economic resources and the decentralized infrastructure of medical facilities [4, 6, 7].

The biosensor technologies that have emerged over recent years and are actively developing serve as innovative platforms for analyzing biomarkers of the infectious process have a high potential to become affordable, fast and reliable in operation, highly specific and sensitive tools for timely and true diagnosis of bacterial and viral diseases [8, 9]. The economic feasibility and ease-of-use of these portable analytical systems are fully consistent with the modern global concept of point-of-care testing (laboratory testing at the site of treatment).

## Modern diagnostic technologies based on the point-of-care concept

In the global practice of infectious disease diagnostics, the point-of-care strategy is becoming increasingly important, based on modern molecular diagnostic technologies, including laboratory testing by the medical personnel at the patient’s bedside or self-monitoring of certain laboratory parameters by patients at home [8, 9].

**Figure 1:**
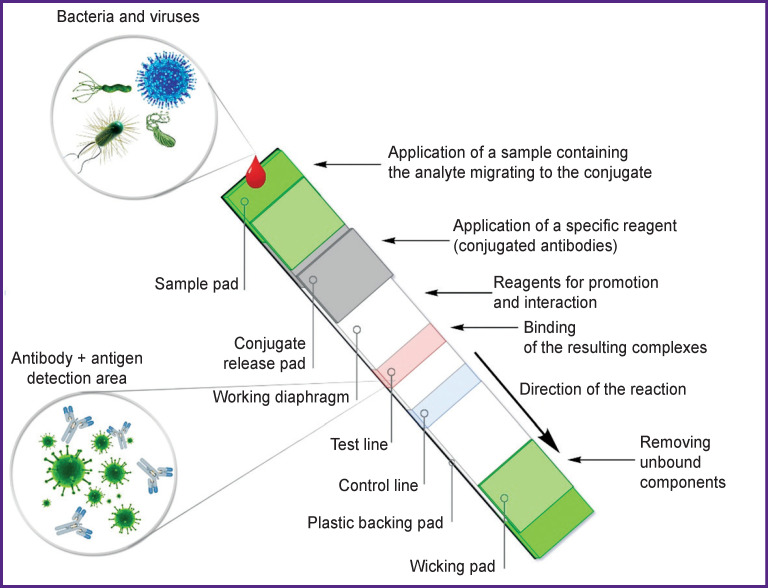
Schematic representation of the lateral-flow immunoassay mechanism The sample containing the test antigen (analyte) is applied to the sample application pad and migrates to the conjugate. The specific reagent with the target analyte migrates to the test line, where they form a complex with antibodies (source: authors)

The diagnostic platforms-precursors of this innovative strategy include qualitative and semi-quantitative test systems for identifying specific antigens [9] and antibodies [10], as well as gene amplification products [11, 12], based on latex agglutination, immunochromatography, and variations of lateral flow immunoassay (lateral flow assay, LFA; and lateral flow immunoassay, LFIA), which have not lost their significance for the diagnosis of infectious diseases these days [10, 11, 13–16] (Figure 1).

For example, in their recent study, Jørgensen et al. [17] successfully tested the first commercial combined test for the detection of *Streptococcus pneumoniae* and *Legionella pneumophila* urinary antigens with the LFIA method. This has increased the popularity of the universal technology that is equally effective in the sandwich analysis format for both high-molecular antigens of microorganisms and antibodies to them in biosubstrates and low-molecular analytes [16, 18–20].

Today, the test systems based on the LFIA method in both standard and multiplex formats make up the most part of the global segment of rapid laboratory diagnostics [14, 16, 18, 19]. However, despite the obvious attractiveness of LFA and LFIA methods, significant disadvantages of immunoassay hinder the expansion of practical use of these diagnostic platforms in the diagnosis of bacterial and viral infections (Table 1).

**Table 1:** Advantages and disadvantages of the test-systems on the lateral-flow immunoassay platform

Advantages	Disadvantages	References
Inexpensive, fast, and easy-to-use tests; long shelf-life of the test systems	Applicable only for primary screening and require confirmation of positive results by independent methods	[13, 17]
No special temperature conditions for storage are required	Special equipment (scanners, reflectometers, CCD cameras) and software are required to obtain quantitative results	[16, 20]
No special equipment is required	Technological improvement of the method increases the cost and duration of the analysis	[14, 18]
Qualified personnel is not required; can be operated by general practitioners or home patients	In the competitive format, the response negatively correlates with concentration	[15, 19, 21]
The visual result is clear and readily visible	Potential technical errors in sample application may affect accuracy and reproducibility of the result	[18, 19, 21]
Tests are usually sold in kits with a set of all the items necessary to perform a test	An increase in the sensitivity of tests is associated with the use of gold and silver nanoparticles or an enzyme, which limits shelf-life, increases the cost of the analysis, and breaks the one-step mode of the test	[15, 19, 21]
Possible increase in the sensitivity of the test systems when using plasmon resonance, surface-enhanced Raman scattering (SERS), chemiluminescent or fluorescent labels	A test sample must be in the form of a solution Pre-dissolution of dry samples is mandatory Low analyte content in the solution requires sample concentrating	[13, 15–17, 21] [13–15, 18, 21]

## Biosensor technologies

Thanks to the impressive advances in molecular biology and nanotechnology since the beginning of the twenty-first century, biosensors are becoming increasingly developed and used for diagnosis both *in vitro* and *in vivo*. Over the past 10–15 years, the research and development of these highly selective analytical devices has become a popular and most actively developing biotechnological trend, the most attractive alternative to lateral-flow immunoassay methods [22, 23].

With putting these fast-acting, sensitive, and inexpensive autonomous sensors into the laboratory practice, the most advanced achievements in various biotechnological fields, including healthcare are expected to appear in the near future. Biosensors have shown great potential for use in medical laboratory diagnostics, as well as a tool for immediate detection in real-time of several markers of bacterial and viral infections. The microorganisms consist of a wide range of macromolecules with electrochemically active groups that can react with free electrons of the electrode surface [22, 23]. Controlling these processes with the use of physical and chemical methods enables to detect and study infectious pathogens. In this case, changes in temperature and pH are used as additional analytical information. Due to these properties, biosensors are considered as a powerful diagnostic technology in the point-of-care strategy for detecting infectious diseases at the initial stage, monitoring the development of the pathological process, and carrying out epidemiological studies [22, 24, 25].

Moreover, due to the selective capability of the devices based on the advances in modern nanoelectronics to receive and convert biosignals, it has become possible to conduct quantitative monitoring of the infectious process [22, 24–27]. For example, by now, we have accumulated experience in using electrochemical biosensors to monitor biofilm formation in real time [22, 25, 26] and other dormant forms of bacteria [27], sepsis development [11, 12, 28, 29], spore formation [30–32].

We shall consider the main types of biosensors and the most common methods of biosensing, as well as modern achievements in designing these analytical devices. Impressive results have been obtained in the use of biosensors in various fields of biology, ecology, toxicology, parasitology, criminology, medicine, and microbiology over the recent years. However, this review will focus on the prospects for using modern analytical devices in the laboratory diagnostics of infectious diseases.

## Main types of biosensors and their functioning

The study of the molecular basis of pathogenicity of microorganisms as well as the search and development of highly effective and sensitive methods for the identification of pathogenic microorganisms have always been the focus of attention for researchers. Besides, timely laboratory diagnosis is the key to successful treatment of infections, as well as prevention of the occurrence and spread of epidemics. For the diagnosis of infectious diseases, monitoring and early detection of the markers of infectious agents are essential. Thus, the development and practical use of biosensors based on modern advances in molecular biology and nanotechnologies fully comply with the current goals of global healthcare and are aimed at solving stated problems [22–24, 26].

According to the definition of IUPAC (International Union of Pure and Applied Chemistry) [33], biosensors are integrated autonomous devices that represent quantitative or semi-quantitative analytical information about the target analyte using a biological element of biorecognition (bioreceptor) located in spatial contact with the transducer. Biosensors do not require additional reagents and differ from other analytical systems in this respect. Thus, biosensors are portable analytical devices equipped with biological elements that can potentially control the biochemical parameters of physiological and pathological processes that are not accessible to modern analytical tools [25]. These are unique detectors the action of which is based on the specific interaction of biomolecules and bioreceptors and which are used to detect and identify the minimum concentrations of various analytes [23, 25, 26]. When a bioreceptor binds to a target molecule, the ligand-receptor interaction is converted into an optical, spectral, or electrochemical signal the power of which is proportional to the analyte concentration [26].

The idea of developing biosensors appeared more than half a century ago, and putting it into practice started [34, 35]. The very first sensors were designed to quantify relatively simple biochemical analytes (glucose, myoglobin, urea, cholesterol, prothrombin). The glucose meters of various modifications for monitoring patients’ blood glucose levels at home are an example of the most common modern biosensors. The glucose oxidase or glucose dehydrogenase enzymes are used as a biosensing component. They are immobilized on the surface of the electrode and break down glucose. The products of enzymatic reactions are converted into a physicochemical signal [25, 26, 36]. However, only in the recent years, thanks to the integration of nanoelectronics and biochemistry, the idea of biosensing has been widely developed. There is a wide range of biosensors that use biological materials to recognize certain biomarkers of the infectious process, which are quantified using optical, micromechanical, interferometric, and other alternative types of transducers [7, 21, 25, 26, 37].

In the modern world, a considerable growth of interest is observed in biosensor technologies, which are rightly considered to be one of the rising trends in the scientific and technical sphere [30, 38–40]. According to the experts’ forecasts, in 10–15 years the market for these analytical devices will exceed $ 70 billion [25, 26]. Most of them are focused on conducting laboratory studies of biofluids for early and accurate quantitative rapid identification of the molecular markers of myocardial infarction, diabetes, sepsis, as well as identification of the markers of parasitic and infectious diseases [25, 26, 41, 42].

**Figure 2:**
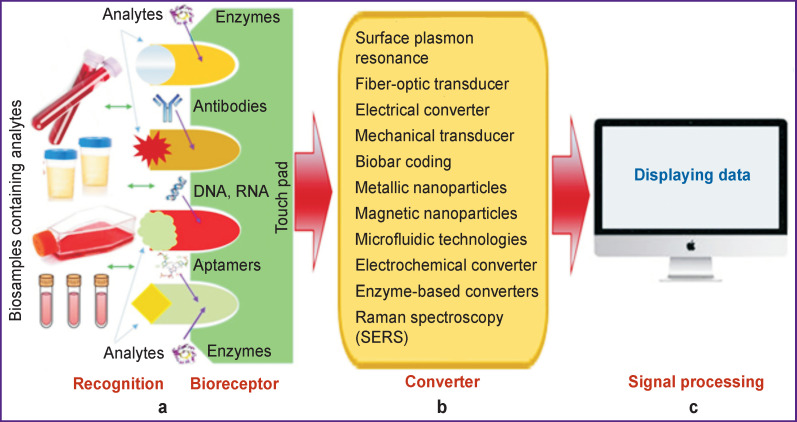
Schematic diagram of the biosensor design The main parts are a bioreceptor with recognition elements for selective (specific) binding of target analytes contained in biosubstrates (a); converter (b); and also an electronic device for signal processing and data display (c) (source: authors)

Structurally, biosensors are a complex consisting of three main functional segments:

1. a bioreceptor (a biosensing component) with the elements on the sensor plate for recognizing target analytes (target molecules) contained in the biosubstrates;
2. a transducer operating on physical and chemical principles (electrochemical, spectroscopic, or optical);
3. an electronic device for signal processing, recording, and displaying data in a convenient (analog or digital) form for the researcher (Figure 2).

The analysis of the literature demonstrates interest in the development and use of these analytical devices in ecology, toxicology, agriculture, biosafety, and medicine, including the clinical diagnosis of infectious diseases and septic conditions. Over the decades of the development of the biosensing technology, a large number of structurally different sensors and attempts to systematize them have been proposed [25, 26, 41–43].

Currently, the biosensor classification is based on the nature of the biochemical component used, the analytical tasks to be solved, the type of signal transducer, the intended application field, and the generated signal. The technical strategies conditioning the ways of further detection (transduction) of the signal/event determine the basic principle of differentiation of the analytical devices [43, 44].

**Figure 3:**
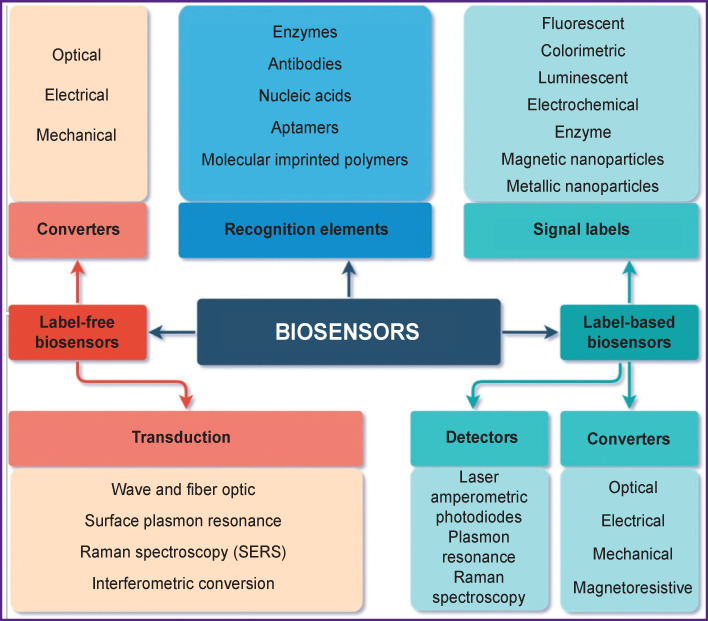
Classification of biosensors based on constructive strategies of detection methods: labeled and label-free biosensors (adapted from [33])

According to this principle, in one of the proposed IUPAC classifications [33], all biosensors are divided into two large groups based on technical strategies and design differences used in the development of detection methods by analytical devices: label-free and label-based ones (Figure 3).

The recognition elements (bioreceptors) are common to all types of biosensors used in biomedical diagnostics: immunoglobulins (antibodies), enzymes (or microbial cell homogenates), nucleic acids (DNA, RNA, and PNA — peptide nucleic acids) [45–47], microbial cells (microorganisms) [5, 42, 44], and aptamers (short DNA and RNA oligonucleotides that can specifically bind to specific targets-molecules) [3, 40, 48]. These receptor biomolecules with concentrations ranging from 1 to 5 mg/mm^2^ are immobilized on a solid sensor substrate (matrix) by covalent binding or biotin-avidin interaction. They serve for selective binding and identification of target analytes (ligands) in biological fluids (whole blood, serum, plasma, urine, saliva, liquor, tissue and cell cultures extracts) [3, 5, 49–51].

When designing highly sensitive biosensors, the correct choice of the matrix and conditions for immobilization of bioreceptors is of key importance. When using non-covalent binding, the receptor is retained on the sensor substrate of the transducer due to electrostatic, van der Waals, or ion interactions that hold the biomolecules fairly firmly. The main advantage of this type of immobilization is that the matrix does not affect the biological properties of the receptor [25, 26]. With covalent binding to the surface of the sensor matrix of the transducer, the biomolecules are held firmly, which prevents them from leaching out of the matrix, and this is of key importance when designing a reusable biosensor [40, 42, 52, 53].

The new-generation devices more often comprise nanomaterials, the unique catalytic efficiency and adsorption properties of which are ensured by the optimal physical and chemical characteristics of the sensor substrate surface [26, 40, 54]. At the same time, neither bioreceptors nor analytes undergo conformational changes and loss of biological activity, which ultimately ensures effective interaction of the ligand with the receptor, which is transmitted as a specific equivalent amplified signal [25, 26, 54, 55].

The mechanism of transmitting a ligand-receptor interaction signal and its transduction is another important functional element of biosensors. Transmission is carried out with electrodes (gold, silver, platinum, mercury, and others with various surface modifications) and graphite pastes [25, 53, 56, 57]. This biochemical process is detected and converted into quantitatively detectable physical parameters with a certain type of physical and chemical converters that provide optical (responding to changes in physical and optical parameters), piezoelectric (quartz crystal microbalance technology), electrochemical (operating on the principle of measuring electric current) or micromechanical signals that are processed by the processor and analyzed at the output [26, 54–56] (see Figure 3).

The principle of operation of bioreceptors can also be represented as three consecutive stages: recognition of the target ligand in the biosubstrate by a specific bioelement located on the touch panel; transduction of information about the biochemical reaction into the form of an electrochemical signal; transduction of this signal into a form convenient for reading or processing by the researcher [26, 51, 55, 56].

For example, in the biosensors, where the enzymes immobilized on the sensor plates serve as a recognition element, the substrates from the biomaterial in the presence of catalysts enter a biochemical reaction with them. The resulting product is determined with the aid of an electrode that transduces the biochemical reaction into an electrochemical signal, the magnitude of which is proportional to the amount of substrate in the studied biomaterial [22, 57, 58].

In recent decades, the key task of interdisciplinary research into the design of modern biosensors (in fact, representing the first generation of bioelectronic devices) has been to improve the parameters of close interaction of biochemical and physical functional elements in order to increase their sensitivity, selectivity, and reduce the detection limits of target analytes [40, 59, 60]. These characteristics of the analytical systems are of prime importance in the diagnosis of infectious diseases.

The progress in the development of biosensor diagnostics of bacterial and viral infections has been achieved mainly due to modern improvements in the methods used for the identification of specific markers [22, 61–64].

Together with the already used analytical devices, where immunoglobulins and enzymes in the form of bacterial homogenates are applied as bioreceptors, in recent years, whole-cell microbial biosensors have been introduced, in which live natural or engineered microorganisms (for example, *Escherichia coli* or *Staphylococcus aureus*) integrated on the sensory substrate assimilate target organic compounds from biosubstrates (for example, antibodies from blood serum), they themselves acting as a sensitive mechanism [25, 61, 64]. In this case, a positive respond to the promoter of the target molecule after its transport through the cell membrane and diffusion inside the bacterial cell causes the expression of the reporter gene, which is recorded as a quantitative response using optical [7, 20, 23, 63, 64] or electrochemical signals [45, 46, 65].

**Figure 4:**
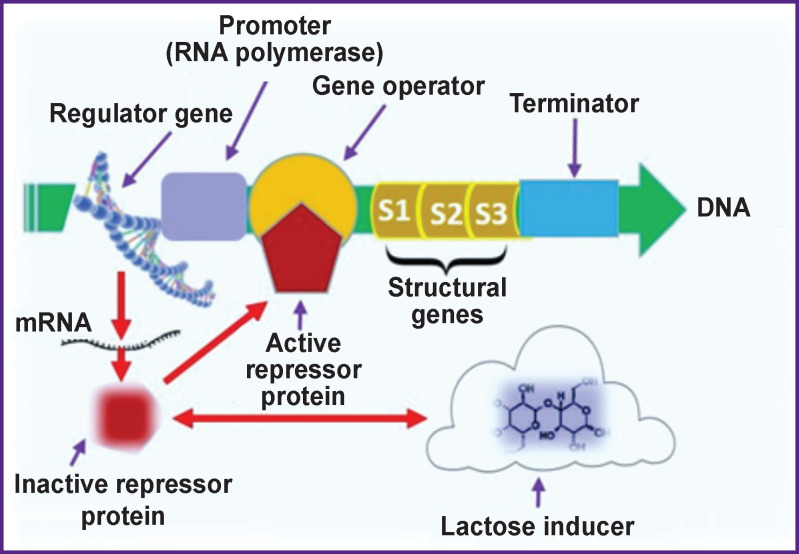
Functional diagram of the lactose *lac*-operon of *E. coli* in microbial biosensors (source: authors)

The use of reporter genes to identify factors that trigger genetic response in living microorganisms was proposed in the middle of the last century [34, 35] when the functioning of the lactose operon of *E. coli* (*lac*-operon) and its relationship with the patterns of metabolism and growth of microbes were described. These fundamental studies were confirmed in subsequent years by studying the role and structure of DNA and other reporter genes, such as *xylE* and *tfdA*, which are currently used actively as a biophysical model for environmental research [26, 66, 67] (Figure 4).

Liu et al. [60] reported on designing a biosensor that uses synthetic antimicrobial peptides as new recognition bioreceptors. The proposed analytical device in combination with the impedance recognition method allowed rapid and quantitative identification of bacterial pathogens in biosubstrates (*E. coli*, *S. aureus*, *Pseudomonas aeruginosa*, and *Staphylococcus epidermidis*) with concentrations ranging from 10^2^ CFU/ml. Besides, this sensor enabled the differentiation between live bacteria and dead ones.

Another group of researchers proposed a biosensor design for highly sensitive and rapid identification of *S. aureus*, where a bacteriophage with a detection range of 4·10^8^ CFU/ml was used as a receptor [68].

Further improvement of microbial biosensors has been due to the emergence of regulatory biosensing associated with advances in molecular genetic technologies and the discovery of new mechanisms for detecting various extracellular and intracellular signals, as well as their subsequent optical and electrochemical transduction [45, 46, 69, 70]. The development of new technologies and advances in synthetic biology resulted in the appearance of biosensors with recombinant nucleic acid receptors and aptamers which are successfully used for diagnosing infections [5, 40, 71, 72]. These technologies provided an increase in the sensitivity of these analytical devices by allosteric regulation of the metabolic signaling pathways of microorganisms, aimed at selective detection of specific biomarkers — small molecules of microbial origin [40, 45, 69–72].

## Label-free biosensors

With the development of modern technologies in infectious disease diagnostics and epidemiology, label-free biosensors have become increasingly more widespread. They enable screening of intermolecular interactions and cellular reactions, provide detailed information about the selectivity of bacterial exotoxins and the specificity of antimicrobial agents, the interaction of antigen with antibody, as well as the kinetics of the inflammatory process, immunological and serological reactions [60, 73].

Currently, there is a wide range of analytical devices for analyzing biospecific ligand-receptor interactions in label-free biosensors. In these highly sensitive and functional systems, the binding reactions of the target analyte to the bioreceptor can be studied without the use of any enzyme, radioactive, or fluorescent labels [11, 61, 74, 75]. Such biosensors do not need expensive reagents and markers, which ensures their cost-effectiveness. These analytical systems are capable of monitoring the reactions of ligand-receptor interaction that occur when target analytes bind to molecular elements immobilized on the sensor substrate (antibodies, enzymes, nucleic acids, aptamers) [61, 62, 76, 77].

This type of biosensor requires only one recognition element, which simplifies the analysis scheme, reduces its duration, and the cost of reagents. The current generation of label-free biosensors allows quantitative measurements of biomolecular reaction products in real time, which makes it possible to perform continuous data recording that enables kinetic monitoring of the parameters of the recognition process in ligand-receptor interactions [27, 78].

An important advantage of using label-free biosensors is that the target analytes are detected in their natural form, without labeling or chemical modification, which means that they can be saved for further analysis (Table 2).

**Table 2:** Advantages of modern label-free biosensors over similar analytical label-based devices

Advantages	References
Simplified pattern of analysis	[3, 46, 48, 73, 79, 80]
Shortened analysis duration (rapid response time)	[7, 78, 81, 82]
Lower analysis cost	[7, 59, 83, 84]
Reduced consumption of organic solvents	[30, 61, 77, 85]
Portability and small dimensions	[30, 40, 71, 80]
No qualified medical personnel required	[3, 7, 36, 61, 80, 82, 85]
Opportunity of quantitative measuring of biomolecules in the real-time mode	[25, 26, 77, 82, 84]
Detection of target analytes in natural forms, without modifications and labels	[22, 30, 71, 81, 83]
High sensitivity	[22, 25, 26, 40, 61, 80, 82]
Direct measurement of analytes	[40, 48, 61, 83]
Opportunity of detecting small molecules	[3, 7, 25, 26, 40, 78]
Opportunity of multiplexing	[59, 61, 73, 85]
Access to kinetic and thermodynamic parameters	[22, 26, 36, 80, 83]

In recent decades, numerous studies have been conducted to develop new types of receptors [60, 68, 73] and recognition methods in label-free biosensors that can generate a signal directly after binding to a recognition element. In this context, there have been proposed many physical and chemical types of transducers that convert the results of bioreceptor binding of targets (for example, an increase in mass, resistivity, and surface refractive indices), which are recognized in various ways [64, 70, 73, 77, 86].

The optical, (piezo)electrical, or (micro)mechanical transducers are among the promising methods for recognizing ligand-receptor interaction signals in label-free biosensors used for the diagnosis of various infectious diseases. These biosensing methods are enhanced by surface plasmon resonance (SPR) [78], surface Raman spectrometry (SERS) [36, 38, 39], quartz crystal microbalance [86], and microcantilever sensors [78, 87, 88].

The biosensors with optical transducers, considered as the main tools for signal perception, are one of the most powerful detection and analysis tools widely used in biomedical research and practical medicine [8, 9, 72, 88, 89]. These transducers are based on measuring changes in optical properties in the presence of the analyte, such as absorption, reflectivity, radiation, or interferometric pattern, which can be detected by a photodetector. They are immune to electromagnetic interference, can perform remote sensing, and have a number of advantages, including high sensitivity, direct real-time measurement, and multiplexing (simultaneous detection of multiple analytes). The microbial biosensors that detect interactions between microorganisms and target ligands are no exception [5, 42–44, 54].

**Figure 5:**
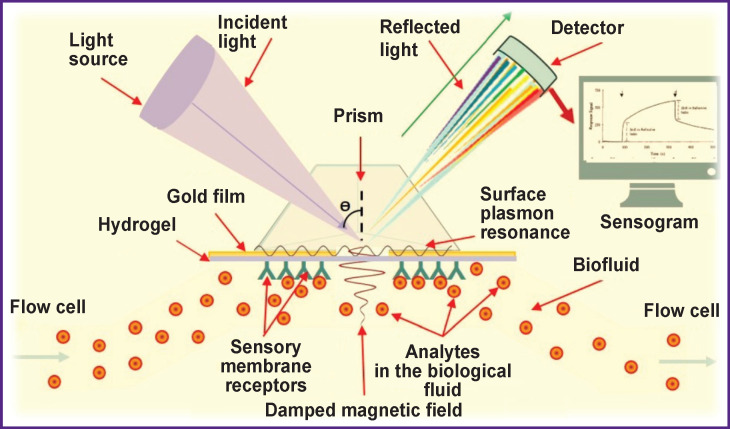
Modern transduction systems in optical biosensors are based on the effect of surface plasmon resonance and damped electromagnetic field (source: authors)

Due to a variety of detection methods using optical transducers in label-free biosensors, the authors of the review limited themselves to the devices that have proved successful in the detection of infectious disease pathogens. The cutting-edge technologies in the design of label-free optical biosensors with the focus on the diagnosis of bacterial infections are associated with the development of modern methods of transduction (fiber-optical and damped electromagnetic field systems, surface plasmon resonance, Raman spectroscopy or interferometry) and new recognition elements (molecular-imprinted polymers) [69, 78, 88, 90–92] (Figure 5).

Among the modern technologies used for the clinical diagnostics of bacterial and viral infections, the development of electrochemical methods of optical biosensing based on nucleic acids presents interest. The recognition elements used in these types of analytical devices include DNA, RNA, PNA, and aptamers. For example, nowadays the sensor technology of DNA hybridization based on electrochemical (impedance spectroscopy) and optical methods which recognizes the complementary target DNA chain of a pathogenic microorganism is gaining popularity, [46, 47, 71, 93, 94]. Due to the ease of use and high sensitivity, electrochemical label-free biosensors have become the most widely used ones for detecting infectious pathogens in recent years (Table 3).

**Table 3:** Examples of modern designs of label-free biosensors for the detection of pathogenic bacteria and viruses

Recognizing bioreceptor	Transduction method	Test models of pathogens (sensitivity)	References
Bacteriophage	Photoluminescence	*S. aureus* (4·10^8^ CFU/ml)	[68, 92]
Antimicrobial peptides	Impedancemetry	*E. coliS. epidermidis* , *S. aureus*, (*P.* 10*aeruginosa*^2^ CFU/ml) ,	[60, 73, 91]
Antibacterial Zn-CuO and nanoparticles graphene oxide Man/MUA-MH/Au*	Impedancemetry Electrochemical impedance spectroscopy	*E. coli*, *S. aureus* (50 CFU/ml); antibacterial effect — 100%, 30 min	[36, 79, 85, 87]
Thiolated G protein on gold electrodes and gold nanoparticles	Cyclic voltammetry Electrochemical impedance spectroscopy	*S. typhimurium* (2.16·10^6^ CFU/ml) *E. coli* (50–10^3^ CFU/ml)	[93]
Enzymes	Electrochemical	*E. coli O157:H7* (150 CFU/ml)	[55, 57, 58]
Nucleic acids (DNA, RNA)	Electrochemical	*S. S. typhimurium aureus* (140 (48 CFU/CFU/ml) ml)	[11, 18, 71]
Nucleic acids (DNA, RNA)	Electrochemical	*S. aureus*, *M. tuberculosis*	[11, 45–47]
Aptamer on gold nanoparticles	Autofluorescence quenching	*S. typhimurium* (48 CFU/ml)	[3, 5, 40]
Monoclonal antibodies	Optical	*S. Listeria enteritidis monocytogenes* (80 CFU/ ml)	[14, 16, 88, 94]
Thiolated aptamer	Impedancemetry	*Shigella dysenteriae*	[8, 95]
Nucleic acids (DNA, RNA)	Electrochemical impedance spectroscopy	*M. tuberculosis*	[62, 88, 96]
Monoclonal antibodies	Surface plasmon resonance	*Enterococcus faecalis* (104–10^8^ CFU/ml)	[86, 90]
Aptamer	Impedancemetry	*Bacillus Bacillus cereus anthracis* (104–10(spores) ^6^ CFU/ml)	[3, 5, 30, 40, 48]
Nucleic acids (DNA, RNA)	Electrochemical Cyclic impedance voltammetry spectroscopy	*Salmonella spp.*	[71]
Enzyme (graphene simulator quantum dots)	Electrochemical	(5 (*Yersinia* milk)–30 *enterocolitica* (serum) CFU/ml)	[48, 80, 97]
Monoclonal (long-term fiber antibodies lattices)	Surface plasmon resonance	*S. aureus* (224 CFU/ml, 30 min)	[69, 78, 90]
Monoclonal antibodies	Visualization	*Salmonella enteritidis* (102–10^8^ CFU/ml)	[81]
Nucleic acids (DNA, aptamer)	Electrochemical	Avian influenza virus H5N1 (AIV)	[45]
Nucleic acids (DNA)	Electrochemical impedance	Zika virus (25.0±1.7 nmol)	[46]
Aptamer (rGO-TiO_2_)	Electrochemical	*Salmonella* (10*enterica*–10^8^ CFU/, *typhimurium* ml)	[80]
Nucleic acids (DNA)	Piezoelectric	*Clostridium* specificity *difficile* (sensitivity — 95%) — 95%,	[85]
Monoclonal antibodies	Surface plasmon resonance	*M. tuberculosis* (102–10^6^ CFU/ml)	[88, 96]
Aptamer	Fluorescent	*Salmonella typhimurium* (6**·**10 CFU/ml)	[82]
Monoclonal antibodies	Potentiometry	*Salmonella typhimurium* (10^6^ CFU/ml)	[84]
Nucleic acids (DNA)	Electrochemical impedance	*M. tuberculosis* (102–10^6^ CFU/ml)	[47]
Aptamer (RNA)	Fluorescent	*S. aureus* (102–10^6^ CFU/ml)	[3]

* Man/MUA-MH/Au — mannose/11-mercaptoundecanoic acid/6-mercapto-hexanol/gold.

The appearance of aptamer-based biosensors (aptasensors) and recombinant nucleic acids as recognition elements has resulted from the development of new technologies and advances in synthetic aptamer biology [3, 40, 48]. These types of analytical devices are very promising due to the high specificity and stability of nucleic receptors, low cost, and the potential for developing various sensor platforms [40, 48].

For example, in a recent study by Sheng et al. [3], they reported the creation of a label-free biosensor with an RNA aptamer that allows rapid quantitative detection of food pathogens. In the proposed aptasensor the RNA-aptamer acts as “antibodies against nucleic acids” of target microorganisms. The oligonucleotide nature of aptamers makes it possible to amplify or chemically synthesize a desired pool with a high frequency and in any quantity, which makes it possible to create highly specific homogeneous sensors providing accurate quantitative detection of pathogen nucleic chains [50, 51, 80, 82, 95].

The authors [3] demonstrated the effective and rapid detection of *S. aureus*, selected as the target pathogen for their aptosensor in food and water. The quantitative assessment of ligand-receptor interaction in the proposed biosensor variant was performed with specific fluorescent dyes for nucleic acids (Sybr Gold and Sybr Green I). It is noteworthy that rapid detection of the pathogen did not require special sample preparation (purification, enrichment).

These modern innovations make optical biosensors more versatile than other types of sensor technologies [62, 72, 74, 76]. They make it possible not only to quantify low-molecular organic molecules (less than 1000 Da), but also to conduct early diagnostics, quantitative analysis of the course of infectious diseases, as well as to carry out epidemiological monitoring [98, 99].

In optical biosensors, the use of the transduction method based on the surface plasmon resonance effect and the phenomenon of total internal light reflection is common [69, 78, 84, 90]. This effect occurs when the angle of incidence is beyond the critical angle with the change of refractive index that occurs on ligand binding to the receptor and increasing the substance mass on the touch sensor pad, and in the reverse process — on reducing the mass-dissociation of the ligand-receptor complex. In this case, an electromagnetic vanishing wave occurs, which, when damped, penetrates the medium with a lower refractive index and creates a so-called damped electromagnetic field (see Figure 5).

If gold (or silver) is sprayed on a hydrogel plate, then another phenomenon occurs associated with the presence of free electrons in this metal(s), which are exposed to the alternating electric field when illuminated. These electrons can collectively vibrate and resonate, adjusting to the frequency of incident light (these electron vibrations in precious metal nanoparticles are called plasmonic ones [61, 62, 69, 87]), and the unique properties of nanometals increase the sensitivity and selectivity of biosensors [71, 90, 95]. Thus, plasmon resonance significantly increases the damped electromagnetic field, reduces the intensity of reflected plane-polarized light, and also allows detecting target analytes in very low concentrations in various biosubstrates without preliminary sample preparation [71, 80, 81, 95].

Among the family of optical label-free biosensors, SPR is one of the most accessible, developed, and most successfully used technologies in recent years for the diagnostics of infectious diseases and septic conditions. This is due to the high sensitivity and versatility of this type of sensors, which also allow real-time detection and direct measurement of the kinetics of molecular ligand-receptor interaction [71, 80]. For example, in recent years, a number of researchers [61, 62, 78, 88, 96] have proposed SPR-based biosensors for detecting and monitoring urinal *M. tuberculosis* biomolecules and non-tuberculosis CFP10 and MPT64 mycobacteria, which, among numerous mycobacteria antigens, interact most strongly with related anti-CFP10 and anti-MPT64 antibodies on the immunosensor matrix.

The only drawback of optical label-free biosensors that use prismatic light refraction and SPR effects is their relatively large size, which is incompatible with mobile use in the point-of-care mode [80, 81]. Therefore, a promising alternative to this type of optical sensors is fiber-optic biosensors, which are perfectly suitable for designing miniature portable devices, have a low cost, and have successfully proved themselves for clinical diagnostics [80].

Label-free optical biosensors have been consistently used for characterization and screening of molecular interactions in clinical laboratories. For example, Golichenari et al. [61] presented a detailed overview of successful uses of the most promising optical label-free biosensors for rapid, highly effective, and accessible detection and quantification of *M. tuberculosis*, mycobacterial proteins, and IFN-γ cytokine as the most important markers in the early diagnosis of tuberculosis.

The majority of modern biosensors intended for the detection of infectious pathogens are based on electrochemical conversion of the signal. These sensors are based on measuring changes in the current, electrochemical potential, and impedancemetry as a means of conversion of biochemical reactions [46, 47, 85]. When designing modern electrochemical biosensors for detecting bacterial pathogens, particular attention is paid to bioelectrodes. The most widely used materials are thin polymer films, nanostructured metal oxides, self-organizing monolayers of organic molecules (SAM), and carbon nanostructures (nanotubes, fullerenes, and graphenes) [48]. For example, SAM are considered to be an ideal material for the immobilization of nucleic acids when designing biosensors for detecting bacteria [48, 80, 97].

The advantages of these devices include portability and simple measuring equipment. Together with their cost-effectiveness, high sensitivity, and large linearity detection range, the electrochemical sensors are capable to work with small sample volumes. Moreover, the result is not affected by sample turbidity, unlike the optical methods based on spectroscopic transduction [74, 100].

## Conclusion

The analysis of the research results published in recent years has shown that the development of molecular biology methods with the aid of nanotechnology opens up broad prospects for designing new biosensor platforms with highly efficient, highly sensitive, and highly selective detection of molecular infectious biomarkers.

Medical biosensors as a new type of diagnostic tools are at the initial stage of their development. However, the first decades of practical application of these analytical devices in healthcare have shown their absolute attractiveness and prospects for detecting bacterial and viral pathogens. The development and implementation of biosensor technologies in clinical laboratory practice is a modern non-alternative strategy for reducing infectious diseases in the regions with a low level of healthcare, where cheap and highly effective diagnostics can play a key role in timely verification of pathogens. The development of inexpensive and affordable analytical devices for clinical diagnostics with lower detection limits for pathogens is necessary due to the importance of diagnosing infectious diseases at the preclinical stage. In this regard, successful experiments in designing and using biosensors for the detection of spore and uncultivated bacteria with an assessment of their viability pose interest [31, 32, 79, 81, 87, 101].

This review focuses on a large group of biosensors that are more available and do not need labels for the reproduced signal, but have rather complex transduction systems. However, the future is for simpler and more portable diagnostic analytical systems that do not require complex conversion platforms (surface plasmon resonance and surface Raman spectrometry) and can detect multiple pathogens simultaneously on the base of multiplex analysis. Such biosensors will be able to solve the global problem of effective control of infectious diseases.

Modern trends in the development of biosensor medical technologies are associated with the development of new materials for designing transducers and conditions for more effective ligand-receptor interaction. The prospects for expanding the practical applications of biosensor technologies are related to clinical diagnostics which meets the requirements of personalized medicine, and are equally attractive for doctors and patients, particularly when verifying pathogens of infectious diseases.

Moreover, one of the key modern trends in the development of clinical laboratory diagnostics is non-invasive testing, which does not involve blood sampling. In this regard, highly sensitive, miniature, and portable medical biosensors with their capability to continuously monitor *in vivo* metabolites, drugs and molecular markers of the infectious process will soon play a leading role.
